# S2k-Guideline – Standardized application of the nasal and conjunctival provocation test on allergic diseases of the upper airways of the German Society for Allergology and Clinical Immunology (DGAKI), German Society of Oto-Rhino-Laryngology, Head and Neck Surgery (DGHNO-KHC), German Dermatological Society (DDG), German Society of Pediatrics and Adolescent Medicine (DGKJ), German Society of Ophthalmology (DOG), German Respiratory Society (DGP), German Society for Occupational and Environmental Medicine (DGAUM), German Society for Pediatric Allergology and Environmental Medicine (GPA), German Society for Pediatric Pulmonology (GPP), Ärzteverband Deutscher Allergologen (AeDA), and German Professional Association of Otorhinolaryngologists (BVHNO)

**DOI:** 10.5414/ALX02647E

**Published:** 2026-09-22

**Authors:** Ulrike Förster-Ruhrmann, Tobias Ankermann, Sven Becker, Petra Bubel, Mandy Cuevas, Moritz Gröger, Ludger Klimek, Lars Lange, Katrin Milger, Philip Maier, Norbert Muelleneisen, Dennis Nowak, Heidi Olze, Uwe Pleyer, Wolfgang Schlenter, Sebastian Schmidt, Antje Schuster, Martin Wagenmann, Bettina Wedi, Oliver Pfaar

**Affiliations:** 1 Department of Otorhinolaryngology, CCM and CVK, Corporate Member of Freie Universität Berlin, Humboldt-Universität zu Berlin, Charité – Universitätsmedizin Berlin, Berlin,; 2 Childrens Hospital, Städtisches Krankenhaus Kiel gGmbH, Kiel,; 3 Department of Otorhinolaryngology, Head and Neck Surgery, University Medical Center, Tübingen,; 4 ENT practice Dr. Bubel, Eisleben,; 5 Faculty of Medicine and University Hospital Carl Gustav Carus, Department of Otorhinolaryngology Head and Neck Surgery, TUD Dresden University of Technology, Dresden,; 6 Department of Oto-Rhino-Laryngology, Head and Neck Surgery, Ludwig Maximilian University of Munich, Munich,; 7 HNO am Maximiliansplatz, Munich,; 8 Center for Rhinology and Allergology, Wiesbaden,; 9 Department of Pediatrics, GFO-Kliniken Bonn, St. Marien-Hospital, Bonn,; 10 Department of Medicine V, University Hospital of the Ludwig Maximilian University Munich, Munich, Germany,; 11 Division of Respiratory Medicine, Department of Internal Medicine, Medical University of Graz, Austria,; 12 Eye Center, Medical Center, Faculty of Medicine, University of Freiburg, Freiburg,; 13 Asthma-Allergiezentrum, Leverkusen,; 14 Institute and Clinic for Occupational, Social and Environmental Medicine, CPC (Comprehensive Pneumology Center) Munich, DZL, German Center for Lung Research, Munich,; 15 Department of Ophthalmology, Charité – Universitätsmedizin Berlin, Freie Universität Berlin and Humboldt- Universität zu Berlin, Berlin,; 16 Langenselbold,; 17 Department of Pediatrics, University Medicine, Greifswald,; 18 Center for Paediatric and Adolescent Medicine, University Medical Center, Düsseldorf,; 19 Department of Otorhinolaryngology, Düsseldorf University Hospital (UKD), Düsseldorf,; 20 Department of Dermatology and Allergy, Comprehensive Allergy Center, Hanover Medical School, Hannover, and; 21 Department of Otorhinolaryngology, Head and Neck Surgery, Section of Rhinology and Allergy, University Hospital Marburg, Philipps-Universität Marburg, Marburg, Germany

**Keywords:** nasal allergen provocation test, conjunctival allergen provocation test, allergic rhinitis, identification causative allergen, rhinomanometer, PNIF

## Abstract

Broad evidence confirms that AR (ICD J30.3) is a chronic disease with high prevalence among children and adults worldwide. Its consequences, both from a public health perspective and at the level of the individual patient, are often underestimated, and the condition is inadequately treated. In addition, AR is often related to comorbidities with additional impact on diseases-severity. Allergen provocation of the easily accessible nasal (nasal allergen provocation test: NPT) or conjunctival mucosa (conjunctival allergen provocation test: CPT) reproduces the allergen-dependent, IgE-mediated allergic reaction under controlled and standardized conditions. These measures are regarded as the diagnostic gold standard, particularly in cases of unclear results in primary allergological tests, enabling clinically relevant identification of the causative allergen. Approved test allergens are critical for ensuring the methodological quality of the NPT. However, their availability is increasingly limited, although adequate quantities of allergen solutions remain indispensable for high-quality nasal allergen testing. Unilateral allergen provocation has been established in the clinical routine for decades in Germany. However, scientific evidence for unilateral application of the test allergen is pending. International position papers differ from this recommendation. The CPT is a simple, safe, and well-tolerated measure. It is primarily used in perennial and, in selected cases, seasonal allergic conjunctivitis. This method is less commonly applied for vernal keratoconjunctivitis and atopic keratoconjunctivitis. It is primarily used to confirm the underlying allergen, particularly when establishing the indication for allergen immunotherapy (AIT). It can also be employed to monitor treatment effects such as e.g., AIT or pharmacotherapy. Although highly sensitive, the procedure of nasal ASA provocation remains a time-consuming procedure. While challenging in children, NPT provides clinically relevant additional diagnostic information on the causes of the allergic symptoms. Allergological diagnostic work-up also includes pulmonary assessment, particularly in patients with established asthma or symptoms suggestive of asthma. Both AAR and PNIF are applied as standard procedures for assessing the outcome of NPT in Germany. In individual cases, other rhinometric methods may also be used.

Published by the German Society for Allergology and Clinical Immunology (DGAKI). Version 2.0. Completion July 15, 2026; Valid until: July 14, 2031

S2k Guideline. Version July 8, Version number 2.0. Registry Number 061-009

## 1. Prevalence of allergic rhinitis (children/adults) 

Allergic rhinitis (AR) is an IgE-mediated early and late reaction following contact with allergens [1, 2]. AR is often accompanied by inflammation of the conjunctiva in the form of allergic conjunctivitis (AC) or allergic rhinoconjunctivitis (ARC) [3, 4]. 

AR is a very common disease in children and adults worldwide (ICD J30.4), with a lifetime prevalence of 2 – 25% in children and up to 46% in adults worldwide [5], and 17 – 28% in Europe [1, 2, 6, 7]. In Germany, the KIGGS study showed a lifetime prevalence of 11% in children and adolescents, while the DEGS1 survey for adults showed a prevalence of 14.8% [8, 9]. Notably, even higher prevalence estimates were observed in the German GEDA cross-sectional study, which reported a prevalence of 20% (195/1,000 inhabitants, ~ 12.5 million) based on self-assessment in adults [10]. Billing data from the Central Institute for Statutory Health Insurance Medical Care shows a prevalence of 7.1% in Germany for 2019. This survey showed a decline in prevalence among children and an increase in the prevalence of allergic rhinitis (AR) among adults between 2010 and 2019 [11]. Secondary morbidity is particularly severe for adults, children, and adolescents with AR, limiting their physical and mental performance and subjective well-being [12, 13, 14]. The disease also imposes a considerable financial burden on healthcare systems [15, 16]. Despite these facts, the disease is often underestimated and inadequately treated because it does not have an immediately serious or fatal course [2, 17]. 

### Conclusion 

Taken together, broad evidence confirms that AR is a chronic disease with high prevalence among children and adults worldwide. The consequences for both healthcare systems and individual patients are often underestimated, and the condition is inadequately treated. In addition, AR is often related to comorbidities with additional impact on diseases-severity. 

## 2. Definition of nasal/conjunctival provocation test (NPT/CPT) 

In a type I allergic reaction of the nasal mucosa or conjunctiva, an environmental allergen cross-links allergen-specific IgE bound to high-affinity IgE receptors on mast cells or basophils, leading to degranulation [18, 19]. The released inflammatory mediators, such as histamine and cytokines, are detectable both locally [20, 21] and systemically [22], triggering the cardinal symptoms of AR or conjunctivitis and, in some cases, systemic symptoms [18, 19]. This allergen-dependent allergic reaction is reproduced under controlled conditions by allergen provocation on the easily accessible nasal mucosa (nasal allergen provocation test: NPT) or conjunctiva (conjunctival allergen provocation test: CPT) [23, 24]. NPT and CPT are also used to monitor the effectiveness of therapies and for dose-finding studies for pharmaceuticals and allergen immunotherapies. They are considered the gold standard in the diagnosis of allergic rhinitis/conjunctivitis, as they can distinguish clinically silent sensitization from clinically relevant allergy [18]. In individual cases, NPT is also used to demonstrate a reaction of the nasal mucosa to a suspected allergen such as where skin tests are negative or not possible, or where there is no serological evidence of allergen-specific IgE antibodies. It is also debated as to whether allergen-specific IgE can be produced exclusively in the nasal mucosa (with a lack of specific IgE detection in the skin and serum) and that the detection of a local nasal allergy in these rare individual cases can only be achieved by means of an NPT [18, 25, 26, 27, 28, 29, 30]. 

The NPT was first described in 1873 by Blackley, who exposed grass pollen directly to the nasal mucosa [31]. In 1958, posterior rhinomanometry was introduced into the NPT [32]. Interest in the NPT grew in the 1970s, and it became increasingly established in routine diagnostics from the 1990s onwards [33]. CPT was first described in 1932 [34] and in more detail in 1959 [35]; its precision was not tested until 1984 by Möller et al. in an initial clinical study [36]. 

In either of the tests, the suspected symptom-triggering allergen (especially aeroallergens, but also medications or occupational substances) is applied to the nasal mucosa or conjunctiva in comparison to a negative control, and the resulting clinical reaction is recorded using subjective and objective parameters [19, 31, 37]. In NPT, the typical nasal symptoms of obstruction and hypersecretion as well as itching and sneezing are recorded, and in CPT, tearing, itching, redness, and oedema (chemosis) are recorded. In addition, cutaneous, bronchial, and systemic reactions are noted [19, 30]. The literature describes various methods and evaluation criteria for NPT and CPT [24]. The European Academy of Allergology and Clinical Immunology (EAACI) published a position paper with the aim of standardizing the NPT and the CPT in the future [30]. No international guideline has been established to date. 

In addition to allergen-specific provocation, non-specific provocation (e.g. with methacholine, histamine, mannitol) is also possible to detect nasal hyperreactivity (analogous to bronchial methacholine provocation to detect bronchial hyperreactivity). Since there is very limited evidence for non-specific nasal provocation testing, its clinical benefit is questionable, and it is not used in routine clinical practice [38]. This guideline addresses only the allergen-specific NPT and CPT. 

### Conclusion 

Allergen provocation of the easily accessible nasal (nasal allergen provocation test: NPT) or conjunctival mucosa (conjunctival allergen provocation test: CPT) reproduces the allergen-dependent, IgE-mediated allergic reaction under controlled and standardized conditions. 

## 3. Indications for NPT/CPT 

Provocation tests play a key role in allergy diagnostics [30]; however, they should not be used routinely. In cases of clinically suspected allergic rhinis/conjunctivitis caused by an inhaled allergen – or allergic bronchial asthma – NPT or CPT may be indicated after completion of basic allergy diagnostics (medical history, skin testing and/or serological allergy diagnostics). Indications for performing NPT or CPT are:

- if the allergy test results do not clearly correspond to the symptoms reported in the patient’s medical history, but clarification of the clinical relevance of an allergen is therapeutically important, for example when establishing the indication for allergen immunotherapy. Unclear findings may occur particularly in cases of local allergic rhin(oconjunctiv)itis [39] but also occur in other everyday clinical situations.
- if sensitization to several seasonal allergens is present, the clinical relevance of which cannot be clearly determined from the patient’s medical history due to the overlap of the respective pollen seasons; provocation testing is indicated when it comes to the correct selection of allergen immunotherapy [40].
- if sensitization to an inhaled allergen has been detected, but the information of the patient does not allow clear conclusions to be drawn about the clinical relevance of the sensitization for the symptoms. This may be the case in particular with perennial rhinitis where, in addition to allergens, numerous non-allergic factors may also contribute to the severity of the clinic picture [41].
- if the relevance of occupational inhalation allergens needs to be demonstrated in the case of occupational health consultations and/or assessments [42].
- in selected cases, when CPT is considered appropriate for potentially severe forms of vernal keratoconjunctivitis (VKC) or atopic keratoconjunctivitis (AKC) [19, 43, 44, 45, 46, 47, 48, 49].
- in addition, NPT can also be used to detect ASA (aspirin) intolerance or NSAID-exacerbated respiratory disease (N-ERD) [50].

In clinical research, NPT and CPT are used:

- to obtain locally produced secretions, the analysis of which in terms of cells and mediators contributes to understanding the pathogenesis of allergic reactions [51].
- for verification of the effectiveness of anti-allergic therapy.

### Conclusion 

NPT/CPT are regarded as the diagnostic gold standard, particularly in cases of unclear primary allergological test results, enabling identification of the clinically relevant causative allergen. 

### Recommendation 

NPT/CPT should be applied as the diagnostic gold standard, particularly in cases of unclear primary allergological test results and if identifying the causal allergen has important clinical implications such as for AIT, pharmacotherapy, or occupational health assessments (strong consensus: agreement of 100%). 

## 4. Contraindications for nasal and conjunctival provocation (NPT/CPT) 

### Absolute contraindications for NPT and CPT

- Condition following severe anaphylactic reactions to the allergen [19, 30, 37];
- Uncontrolled severe asthma or severe COPD (see section 12) [19, 30, 52];
- Other relevant conditions, e.g., unstable coronary heart disease/unstable arterial; hypertension [30].

### Temporary contraindications

- Acute viral or bacterial conjunctivitis, rhinosinusitis; bronchitis/pneumonia;
- Acute exacerbated rhinoconjunctivitis and/or corresponding asthma symptoms;
- Interval between two positive nasal or conjunctival provocations: minimum interval of 1 week;
- Drug withdrawal periods for medication (see section 8);
- Minimum interval of 1 week after vaccination.

### Specific NPT contraindications

- Minimum interval of 6 – 8 weeks after sinus surgery [32, 52].

### Specific CPT contraindications

- Chronic eye diseases such as Sicca syndrome [19];
- Minimum interval of 3 months after eye surgery;
- Minimum interval of 72 hours after removal of contact lenses for CPT [19].

Caution is advised when performing NPT or CPT on young children (see section 11) [52]. Technical difficulties arise with NPT in cases of choanal atresia, septal perforation, or nasal polyps (see section 6.1. and section on technical measurement methods in the Supplemental material). 

## 5. Allergen test solutions 

### The medicinal product definition for test allergens used in provocation testing 

Provocation tests require test allergens that are medicinal products in accordance with § 2 of the German Medicinal Products Act (AMG), as they are used for diagnostic purposes. Allergens have been subject to European law since 1989 (Richtlinie 89/342/EWG –> Directive 89/342/EEC). According to the definition in the current Richtlinie 2001/83/EG –> Directive 2001/83/EC and its implementation in national law (AMG), both test and therapeutic allergens are medicinal products [53]. Since 2005, with the 14th amendment to the AMG, allergens have been classified as finished medicinal products, as is the case for other medicinal products involving an industrial manufacturing step. According to § 4 –> Section 4 (1) of the AMG, finished medicinal products are medicinal products that are manufactured in advance and placed on the market in a package intended for supply to the consumer. One of the most important prerequisites for the “marketing” of finished medicinal products is that they are authorized [54]. A medicinal product is authorized upon application after examination by the competent federal authority if its quality, efficacy, and safety are guaranteed and there is a positive benefit-risk ratio [53, 54]. 

The European Pharmacopoeia (Ph. Eur.) is the official rulebook of quality requirements for nasal and conjunctival provocation test solutions and all other medicinal products in Europe. Different monographs apply depending on the properties and type of application of a product. Since nasal provocation test solutions are based on aqueous extracts produced from the respective starting material, they fall within the scope of the monograph on allergen preparations. The central objective of this monograph is to ensure the identity, content, and reproducible quality of allergen products. Updates to the pharmacopoeia must be implemented by pharmaceutical manufacturers in the production of approved nasal provocation test solutions [55]. 

The “Guideline on Allergen Products: Production and Quality Issues” published by the European Medicines Agency continues to apply. Allergen preparations reflects the requirements of the Ph. Eur. monograph. 

Allergen preparations supplements their regulatory requirements with more detailed information. Both documents have been specifically tailored to the particular challenges associated with allergen products due to their biological origin (e.g. natural variation in protein content and biological activity) [55]. In addition, the requirements of Good Manufacturing Practice (GMP) and Good Distribution Practice (GDP) apply. 

### Availability of diagnostic allergens in Germany 

Changes in European legislation and, above all, a decline in demand, mean that fewer and fewer test allergens are available for nasal provocation testing in Germany. While there were still 354 approvals for provocation test allergens in Germany in 2011, there are currently only 74 (the current status can be found on the Paul Ehrlich Institute website; https://www.pei.de/DE/arzneimittel/allergene/provokationstest/provokationstest-node.html). In addition, only a fraction of the approved test allergens are actually available, as described below [56]. The situation is particularly problematic for occupational type I allergies [57]. The last new approval for a provocation test solution was granted in April 2007. Manufacturers are constantly streamlining and reducing their portfolios, thereby reducing the number of test allergens available for provocation testing. When an authorization is returned, this is published in the next issue of the Federal Gazette, which appears monthly. After publication, there is a 2-year sell-off period for these products [58, 59]. 

Since the relatively high cost is somewhat disproportionate to the sales revenue, no new nasal provocation test solutions have been approved as finished medicinal products in Germany for many years. Instead, most manufacturers have voluntarily withdrawn existing approvals for nasal provocation test solutions on a large scale. The fact that manufacturers have removed numerous approved test allergens from their portfolios and voluntarily returned approvals represents not only a quantitative but also a qualitative loss, as this means that rare but allergologically significant test allergens are increasingly unavailable. A new scientific guideline adopted on June 10, 2025 and coming into force on January 1, 2026 by the Committee for Medicinal Products for Human Use (CHMP), “Guideline on allergen products development for immunotherapy and allergy diagnosis in moderate to low-sized study populations (EMA/CHMP/72790/2024)” [60] aims to simplify the approval process for rare test allergens. Furthermore, the possibility of manufacturing rare test allergens through special pharmacies is also being examined [61]. 

The possibilities of in vivo allergy diagnostics using nasal provocation tests are becoming increasingly limited by the problems described above – even with prick and intradermal tests. It is important to note that the availability of test allergens for routine diagnostics is linked to sufficient sales of test allergens at a reasonable price [62]. 

Allergists will have significantly fewer options for diagnosis, especially of rare allergies, which will impact the treatment of their patients. The medical and economic consequences of this are not yet foreseeable. 

### Conclusion 

Approved test allergens are critically relevant for ensuring the methodological quality of the NPT. However, their availability is increasingly limited although adequate quantities of allergen solutions remain indispensable for high quality nasal allergen testing. 

## 6. Performing nasal provocations 

### 6.1. Test preparation (examination, premises, emergency medication) and examination 

After taking the patient’s medical history, the external nose and face are examined to look for possible signs of an atopic constitution (e.g., Dennie-Morgan folds). This is followed by an anterior rhinoscopy to inspect the front parts of the main nasal cavity to assess nasal secretions, mucosal swelling, crusting as well as pronounced septal deviations, septal perforations, or nasal polyposis, followed by an endoscopic examination if necessary. Anterior rhinoscopy is mandatory before performing an NPT test. 

**Test day/seasonality**

In order to evaluate specific sensitization, determine the indication, and ensure the correct test allergen, a prick test or serological examination of specific IgE should be performed in addition to evaluating the patient’s medical history. Various studies have shown that prior exposure to allergens through a pro-inflammatory stimulus can impair nasal patency (priming) [63, 64, 65, 66, 67]. 

When testing for seasonal allergens, the NPT should therefore be performed ideally no earlier than 4 weeks after the end of the pollen season [67]. 

**Room conditions**

The test room must not be contaminated with other substances (methacholine, test sprays of the allergen spray, etc.). “Test sprays” must be performed in a separate room, under a fume hood, or onto a gauze pad to ensure a valid test result [37]. 

**Staff/Emergency response**

Doctors may delegate the performance of NPT to specially trained nursing/functional staff. Doctors should always be available and assume responsibility for delegating the test procedure. 

Although dermal or pulmonary reactions have been reported extremely rarely following NPT to date, these could occur after nasal allergen provocation, see section 6.3. Therefore, an informed consent should be performed before testing, and it should be documented. 

A basic requirement for performing NPT is an emergency kit with the appropriate equipment located directly on site [68]. All medical staff involved in allergy diagnosis and therapy procedures should be trained in the recognition and management of allergic emergencies. 

In addition to antihistamines (oral/intravenous), corticosteroids (oral/intravenous), short-acting beta-agonists (inhaled), and adrenaline (intramuscular/auto-injector), nasal decongestants are useful for managing the symptoms of an allergic reaction [33]. Algorithms for dealing with allergic emergencies: see anaphylaxis guidelines [69, 70, 71]. 

**Recommendation**

The requirements for safe and reproducible performance of nasal provocation should be followed (strong consensus: agreement of 100%). 

### 6.2. Allergen application (techniques, unilateral vs. bilateral) 

The position paper issued by the Ear, Nose, and Throat (ENT) Department of the German Society for Allergology and Clinical Immunology (DGAKI) emphasizes that the control and allergen solutions must be brought to room temperature before administration [37]. After this, a test spray should be used to check that the nebulizer chamber is filled with the appropriate solution and that the spraying process releases the appropriate amount. 

It was also recommended to provoke the side of the nose with the higher volume flow (as determined by rhinomanometric measurement) [37] and to apply two sprays laterally upwards towards the medial canthus. As an alternative method of allergen application, the authors recommended using filter paper or a pipette. The use of cotton swabs is obsolete. The authors of this guideline state that the reason for strictly unilateral provocation is that bilateral application of the test allergen, which is standard practice in several other European countries, is less standardized than strictly unilateral provocation. However, rhinomanometric volume measurement is also recommended on the opposite side to avoid a possible false test result due to the influence of the nasal cycle (which occurs on both sides). The authors consider the nasal cycle to have a relevant influence if the flow volume on the provoked side decreases by 40%, but the total volume after provocation only decreases disproportionately (by up to 10%) [37]. 

The position paper of the European Academy of Allergology and Clinical Immunology (EAACI) explicitly recommends applying both the control solution and the test allergen to both sides of the nose. One puff is applied to the lower turbinate and one puff to the middle turbinate on each side of the nose [30] (one spray horizontally and one spray diagonally lateral towards the lateral corner of the eye), avoiding the nasal septum to prevent non-specific mechanical irritation [30]. The authors do not specify any advantage of bilateral application over unilateral application [30]. 

As in the German-language position paper, the European recommendation points out that patients must be instructed to take a deep breath before spraying, during administration, the patient should hold their breath and, after administration, breathe out through the nose, to avoid accidental distribution of the test substances into the lower respiratory tract. The authors also favor allergen provocation in the form of a pump-dosing aerosol spray, arguing that this method allows a precise dose of the aerosol (50 µL/spray) to be administered in a targeted manner with minimal non-specific mucosal irritation [30]. 

**Conclusion**

Unilateral allergen provocation has been established in the clinical routine for decades in Germany. However, scientific evidence for the unilateral application of the test allergen is pending. International position papers deviate from this recommendation. 

**Recommendation**

Unilateral nasal provocation should be performed. Bilateral nasal provocation may be performed (consensus: agreement of 90%). 

### 6.3. Performing nasal provocation 

**Timeline of the provocation**

To ensure the reproducibility and specificity of the nasal allergen provocation, a baseline measurement without application of any control or test substance, a control provocation without allergen, and the allergen provocation should be performed sequentially. 

The control provocation is performed with the same solution as the allergen provocation, but without the addition of allergen. It is intended to distinguish between non-specific hyperreactivity and a specific reaction to the allergen. A positive control (e.g., with histamine solution) is not performed. 

**Procedure for provocation[Fig Figure1]**

1. First (baseline) measurement: inspection, symptom scores, rhinomanometry or peak nasal inspiratory flow (PNIF) (alternatively, other methods such as acoustic rhinometry are also possible). Determination of the side to be provoked in a unilateral procedure: the less obstructed side; in PNIF measurements, the measurements are methodically performed endonasally on both sides (Supplemental material).
2. Control provocation: 2 sprays on one side;
3. 10-minute break;
4. Measurement: inspection, symptom scores, rhinomanometry or PNIF;
5. Allergen provocation;
6. 10-minute break
7. Measurement: inspection, symptom scores, rhinomanometry or PNIF;
8. If the measurement result is negative: repeat measurement after another 10 minutes, if necessary, using symptom scores, rhinomanometry or PNIF (see point 4);
9. Follow-up observation.

**Evaluation of results**

To evaluate the results of the provocation test, both objective parameters (rhinomanometry or PNIF, alternatively acoustic rhinometry) and symptoms should be recorded, as several studies have shown that these parameters do not correlate well [30, 72]. 

**Recording symptoms**

Various methods are available for recording nasal symptoms. In principle, ordinal scales (e.g., Total Nasal Symptom Score, Linder, Lebel [73, 74, 75]) or visual analogue scales (VAS) can be used for this purpose. 

Since VAS scales are well evaluated for allergic rhinitis, offer methodological advantages, and are easy to use, they should be used to assess symptoms during nasal provocation. A separate line (100-mm long) on the test sheet should be used for each of the symptoms (1. nasal obstruction, 2. nasal secretion, 3. itching and sneezing), which the patients fill in themselves. A total score can be calculated from the sum of these 3 values. 

Symptoms affecting the bronchi, skin, or systemic reactions occur extremely rarely after correctly performed nasal allergen provocation (in our own patient population, 0.2% in 1,000 nasal provocations) [76, 77]. They therefore do not play a role in the assessment of the reaction in routine clinical practice. 

In justified cases (e.g., to ensure comparability with previous results), the categorical assessment matrix from the 2003 position paper can be applied [37]. 

### Interpretation of results 

The same criteria apply to the evaluation of the positivity of the control provocation as to the allergen provocation. 

A reaction is considered positive if either the objective measurements show a sharp decline (rhinomanometry or PNIF flow decline ≥ 40% compared to the baseline measurement) or the symptoms increase significantly, or if both parameters decline moderately [30]. 

In individual cases, after a > 40% decrease in nasal flow to the control solution without further clinical symptoms, it may be appropriate to continue the provocation and evaluate only the clinical symptoms. A ≥ 40% flow decrease compared to the previous measurement refers to the provoked side in the case of unilateral allergen application (see section 6.2.). 

The following limits for the evaluation of the sum of the 3 VAS values (tVAS) are recommended on the basis of systematic evaluations [78]: moderate increase in symptoms (tVAS): 95 mm, severe increase in symptoms (tVAS): 120 mm. A moderate decrease in nasal flow of ≥ 20% is recommended for rhinomanometric measurement or after PNIF measurement. 

After nasal allergen provocation, in most cases the immediate-phase reaction is followed by a delayed-phase reaction [79]. However, delayed-phase reactions without a preceding immediate phase are not observed. Therefore, clinical observation for diagnostic purposes can be limited to the early reaction. 

## 7. Procedure conjunctival provocation test (CPT) 

The conjunctival allergen provocation test (CPT) is a way of confirming the conjunctival-specific response to a clinically suspected allergen in selected cases of perennial allergic conjunctivitis and, in certain cases, seasonal allergic conjunctivitis [19, 44, 45, 46, 49, 80, 81, 82]. The CPT may also be suitable in selected cases for the potentially more severe forms of vernal keratoconjunctivitis and atopic keratoconjunctivitis [48, 83, 84], see indications in section 3. The CPT is frequently used in clinical studies to test and monitor the therapeutic effect of allergen immunotherapy or anti-allergic treatment [43, 82, 85, 86, 87]. 

### Patient consent 

Before performing the CPT, information about the benefits and risks of the test must be provided, and informed consent must be obtained. 

### Discontinue medication 

Medications that could influence the response to allergen instillation should be discontinued for an appropriate period (Table 2). 

### Allergen test solutions 

Where possible, standardized allergen solutions approved for provocation should be used for CPT. As with NPT, the provocation solutions should be at room temperature before application in order to avoid non-specific reactions [19]. 

In most patients, the provocation solution can be used undiluted. In cases of severe allergy, it may be advisable to start with a 1/100 or 1/10 diluted provocation solution, whereby the specific recommendations in the technical and usage information must be observed. 

### Test procedure 

At the beginning, both eyes are examined for orientation to rule out contraindications such as inflammation. 

Using an Eppendorf pipette or a 1-mL syringe, 0.9% NaCl (20 – 40 µL/ or 1 drop) is applied to one eye as a negative control to rule out non-specific inflammatory reactions. To do this, the lower eyelid is everted, and the control solution is dripped into the lower conjunctival sac (Figure 2). The allergen solution (20 – 40 µL/1 drop) is then applied to the other eye in the same way [19]. 

The conjunctival provocation test is evaluated as follows [43] in Table 1. 

The test procedure is shown in Figure 3. CPT is considered positive if there is a score difference of ≥ 6 between the two eyes. As soon as a positive reaction is recorded, the test is terminated. After a positive CPT, the patient is given antihistamine eye drops and, if necessary, a systemic antihistamine. The patient should be monitored for at least 30 minutes. As an alternative to subjective assessment of the score, photographic documentation has proven helpful in objectifying a CPT reaction and can be used especially in clinical studies [43]. Current developments in CPT assessment include AI-based technologies [47]. [Fig Figure4]

### Conclusion 

The conjunctival provocation test is a simple, safe, and well-tolerated measure. It is primarily used in perennial and, in selected cases, seasonal allergic conjunctivitis. This method is less commonly applied for vernal keratoconjunctivitis and atopic keratoconjunctivitis. It serves mainly for exact confirmation of the underlying allergen, particularly for indication prior to allergen immunotherapy (AIT). It can also be employed to monitor treatment effects such as e.g., AIT or pharmacotherapy. 

### Recommendation 

The conjunctival provocation test should always be performed under controlled and standardized conditions (see flow chart), considering the contraindications listed (consensus: agreement of 86%). 

## 8. False positive and false negative results of NPT and CPT 

### 8.1. Nasal provocation false positive and false negative results 

False positive or negative results may be caused by changes in the responsiveness of the nasal mucosa, errors in performing rhinomanometry (see supplement material) and application of the allergen, prior medication use, or problems in handling the provocation solutions. 

Causes of false positive tests include nasal cycle phenomena, nasal hyperresponsiveness, which is also found in non-allergic and infectious forms of rhinitis, exposure to mucosal irritants, and prior allergen exposure immediately before provocation. In addition, contamination of the air in the examination room with allergens can lead to false positive results. 

Conversely, false-negative results are often observed if medications affecting the mucous membranes have not been discontinued for a sufficient period before testing (Table 2). 

False negative results may occur in cases of low nasal flow at the start of the test, existing anatomical obstacles (e.g., pronounced septal deviation), the use of incorrect or insufficiently concentrated or unusable allergen solutions (expired use-by date, incorrect handling of test solutions), or following surgery with extensive reduction of the swelling tissue in the area of the lower turbinate. 

In the event of a non-specific reaction to the control provocation, pre-treatment with, for example, intranasal corticosteroids may be advisable, followed by a repeat nasal provocation. In case of suspected false positive or false negative test results that can be identified as such, or results that contradict the medical history and diagnosis obtained to date, the provocation should be repeated at intervals of no less than 1 week. 

### 8.2. Conjunctival provocation false positive and false negative results 

Incorrect results may be caused by changes in the responsiveness of the conjunctiva, errors in the preparation of the dilution levels and the application of the allergen, prior use of medication, or problems in handling the provocation solutions. 

False positive test results may occur in cases of existing eye irritation due to current pollen count, previous conjunctival inflammation and eye surgery within the last 3 months, or use of solutions containing phenol or glycerin. 

False-negative test results may be caused by allergen solutions that are too low in concentration or no longer usable, insufficient withdrawal of medications that affect the reactivity of the conjunctiva (Table 2), and contact lenses that have not been removed. 

### Recommendation 

Interpretation of NPT/CPT-test results should consider the main factors leading to false-positive or false-negative outcomes (consensus: agreement of 86%). 

## 9. Special test procedures nasal acetylsalicylic acid (ASA) provocations 

N-ERD (non-steroidal anti-inflammatory drugs exacerbated respiratory disease) is typically characterized by chronic rhinosinusitis with nasal polyposis (CRSwNP), asthma, and NSAID intolerance [88]. The procedure for ASA provocations was established in the EAACI/GA2LEN position paper by Nizankowska-Mogilnicka et al. [50]. ASA provocations are performed nasally, orally, or bronchially [50]. Oral ASA provocations are the gold standard for diagnosis of N-ERD [88]. For nasal ASA provocation, a provocation dose of 16 mg lysine ASA (aspisol) is recommended, which corresponds to an application dose of 80 µL. Endonasal administration is performed using a pipette. Unlike bronchial and oral ASA provocations, patients with uncontrolled asthma can also be provoked nasally. Measurements are taken every 10 minutes (nasal flow; clinical symptoms) until a positive reaction occurs. Studies have shown positive reactions within the first hour (min/max 27 – 55 minutes) [89, 90]. Further information on performing the test can be found in section 6.3. Nasal ASA provocations can be performed on an outpatient basis. The sensitivity of the test is 86.7%, and the specificity is 95.7% [50]. By increasing the test dose from 16 to 25 mg ASA, the sensitivity could be increased to 95% [92]. If there is clear evidence of NSAID use in the patient’s medical history, N-ERD can be diagnosed without an ASA challenge [88]. Nasal ASA challenges are indicated, for example, in cases of unclear NSAID use in the patient’s medical history or in studies to differentiate between N-ERD and non-NERD groups [91]. Bronchopulmonary reactions have been described after nasal ASA provocation (10 – 21%) [89, 90, 92]. Peripheral venous access is mandatory. Emergency medication and a peak flow meter or spirometer should be readily available. 

### Conclusion 

Although highly sensitive, the procedure of nasal ASA provocation remains a time-consuming procedure. 

### Recommendation 

Nasal ASA provocations may be indicated for diagnostic evaluation in cases of unclear history of NSAID intolerance and in research settings by distinguishing between N-ERD and non-N-ERD phenotypes. In selected patients with a history of severe asthma, this method can be applied by experienced rhinologists in close collaboration with pulmonologists (strong consensus: agreement of 100%). 

## 10. Special occupational health aspects 

Occupational health issues relating to nasal provocation testing generally involve determining whether a substance is the cause or a contributing factor in the manifestation of an allergic disease of the upper respiratory tract. This essentially occurs in two scenarios:

- Answering the question of whether an allergen present in the workplace contributes to respiratory symptoms and whether this activity is prognostically unfavorable (e.g., career counselling for workplace exposure to flours and baking additives, animal epithelia, molds, etc.).
- Answering the question (usually in the form of an expert opinion) of whether the medical requirements for recognition of occupational disease (BK) 4301 are met (legal definition of BK 4301: “Obstructive respiratory diseases (including rhinopathy) caused by allergenic substances”) and/or whether preventive measures are to being taken at the expense of the accident insurance provider (§3 of the Occupational Diseases Regulation).

In addition, isolated nasal provocation testing with a suspected workplace allergen may also be helpful if analogous clinical symptoms of the upper and lower respiratory tract are reported, but exposure testing of the lower respiratory tract is not possible due to persistent obstruction and/or the need for treatment of the lower respiratory tract without the possibility of discontinuing anti-obstructive and anti-inflammatory respiratory medication. 

The occupational health indication for nasal or whole-body exposure testing with allergens is based on the following scheme (Figure 5). It should be noted that the desired degree of diagnostic precision may vary in clinical, preventive medicine, (accident) insurance law, and scientific issues, so that the examination effort and the potential examination-related risk to the patient must be weighed differently on a case-by-case basis for the various issues mentioned: 

There are ~ 400 workplace-related allergens that may cause allergic diseases of the upper and lower respiratory tract [95]. The most common ones are listed in (Table 3) [96]. 

In addition to isolated nasal provocation testing with allergens or mixtures of substances containing allergens from the workplace, “whole-body exposure” is often carried out in the occupational medicine context in the absence of contraindications for exposure testing of the lower respiratory tract, in which conjunctival, nasal, and bronchial effects are to be objectified simultaneously. This is done in accordance with the guideline “Workplace-related inhalation test” [94]. Regarding nasal and conjunctival reactions, reference is made to the present guideline. 

As the number of commercially available test allergens has continued to decline in recent years, not only in general but also specifically for testing with occupational allergens – particularly since immunotherapy for occupational allergens is rarely performed – practitioners are unfortunately often forced to resort to provocation with insufficiently standardized mixtures of substances from the workplace. Figure 6 illustrates the practical procedure: 

### Recommendation 

The indication for nasal or, if necessary, workplace-related whole-body exposure to occupational allergens should be carefully evaluated in advance, considering the clinical relevance of the respective measure, potential risks to the patient, and the implications of the results. In addition to these recommendations in NPT, the AWMF guideline “Workplace-related inhalation test” should serve as a reference for exposure testing. In the absence of standardized allergen extracts, exposure using allergens in their native form may be considered (strong consensus: agreement of 100%). 

## 11. Special aspects in pediatric diagnostics 

The various methods of nasal provocation [97, 98, 99] can also be used in children and adolescents, and the results are reproducible. In addition to evaluating symptoms, objective measurement methods should also be used where possible to assess the nasal provocation test (NPT). If a diagnosis based solely on symptoms yields unclear or negative results, a new test cycle with additional rhinomanometry or PNIF should be performed. 

This approach is practically significant, as nasal provocation tests are often used as a safe procedure in pediatric pulmonology as a substitute for bronchial provocation tests (BPT), which are potentially associated with more side effects. In a comparative study, the nasal provocation test (evaluated using the Lebel symptom score, no rhinomanometry) was found to have a sensitivity of 71.9% and a positive predictive value of 89.1%. The authors conclude that an NPT can replace a BPT in most cases [100]. In cases of polysensitization, a nasal provocation test is suitable for discriminating between clinically relevant allergens [101]. 

Practical information: Children are sometimes afraid of procedures they are unfamiliar with. Therefore, the procedure should be discussed with the child and their parents well in advance. Parents should be able to answer their child’s most important questions about the procedure. In addition, the necessary adaptation to the examination conditions should be extended to alleviate any fears and enable a thorough examination. 

Nasal provocation testing is usually successful from around school age and onwards. An attempt can be made to carry out the test on children aged approximately 4 – 6 years. As a rule, there is no necessary indication for nasal provocation in younger children. The aim of nasal provocation is usually to clarify the relevance of sensitization to determine the indication for allergen immunotherapy from the age of 5. This question does not arise at this age. In addition, it can no longer be assumed that the child will be able to cooperate safely: in a correctly performed NPT, the test solution may only be applied nasally after deep inhalation to prevent accidental bronchial deposition. In younger children, correct nasal application of the provocation solution following inhalation cannot be reliably ensured, and accidental bronchial deposition is conceivable. In very young children, too vigorous application of the test solution as a spray can cause pain, so that application of the same dose in drop form should be considered. 

When using anterior rhinomanometry, the flow achieved in younger children with calm nasal breathing may be so low that it cannot be adequately measured by the device. In this situation, children should be asked to breathe in a little deeper. In cases of chronic nasal congestion, it can be challenging to find a time when nasal breathing is unobstructed and nasal provocation is feasible without administering additional medication (compliance with medication withdrawal). 

If rhinomanometry or PNIF cannot be performed, the use of symptom scores such as the Lebel score is helpful, especially in young children (Supplemental material). If there is a clearly positive result after allergen provocation and a negative result in the negative control, the result is quick and sufficiently valid. If there is no clear positive result after allergen provocation, the test is not definitely negative. Provocation with rhinomanometry or PNIF should then be attempted again. Compliance with medication abstinence should be discussed with the child and all caregivers and verified before the test. This is particularly important for children who live alternately in different households (e.g., divorced parents, grandparents, residential group). 

After vaccinations, a minimum interval of 1 week (at least 2 weeks after MMR or influenza A live vaccination) should be observed to avoid any potential influence on the test and to be able to reliably identify possible side effects. 

In the case of conjunctival provocation in children, no further special measures are required beyond providing information and creating a child-friendly atmosphere. 

### Conclusion 

While challenging in children, NPT provides clinically relevant additional diagnostic information on the causes of the allergic symptoms. 

### Recommendation 

In children, nasal provocation assessment may, in some cases, be limited to symptom recording. Therefore, the procedure should be individually adapted for younger children, while from school age onwards the recommendations on procedures in adults can be applied progressively (strong consensus: agreement of 100%). 

## 12. Special aspects of pneumological diagnostics 

### Relevance of nasal provocation testing for the diagnosis of allergic asthma 

In patients with asthma, the clinical relevance of sensitization, especially to perennial allergens such as house dust mites, cannot always be reliably determined on the basis of medical history [102], so that an allergen provocation test may be useful here, especially in cases of divergent results of allergic sensitization [30, 103]. Even though bronchial provocation is the gold standard for asthma [30], it is complex and rarely performed due to the potential risk of triggering a severe asthma attack. In several studies, NPT shows good agreement with a sensitivity of 70 – 80% and a positive predictive value of 89% for predicting a positive bronchial provocation test [100, 104]. Therefore, performing an NPT may be justified as a simpler and safer method, with subsequent bronchial provocation only being necessary in cases that remain unclear. 

### Relevance of asthma for the safety of NPT 

Pulmonary diseases, in particular severe and uncontrolled asthma (see [105, 106] for definition), but also other pulmonary diseases associated with severe restrictions in lung function, such as severe COPD, are contraindications for NPT [30]. If the applied allergen is accidentally inhaled, airway obstruction may occur, especially in patients with known asthma. However, this can be mitigated by administering an inhaled beta-mimetic before nasal provocation. Patients with asthma should have good asthma symptom control and no exacerbation when undergoing NPT and should have taken their inhaled medication. In the case of uncontrolled asthma, preliminary pulmonological diagnostics are recommended to optimize therapy; in the case of partial or good symptom control, NPT can be performed immediately. Asthma symptom control can be easily assessed using 4 questions (Supplemental material: 4 questions according to GINA [106] or see the following link for the ACT score: https://www.asthmacontroltest.com/de-de/welcome/). 

### Conclusion 

Allergological diagnostic pattern also include the pulmonary assessment, in particular in patients with established asthma or symptoms suggestive of asthma. 

### Recommendation 

In cases of uncontrolled asthma symptoms, pulmonological consultation should be carried out before NPT (strong consensus: agreement of 100%). 

## 13. Hygiene regulations 

Nasal and conjunctival provocations (NPT/CPT) are carried out in accordance with current hygiene regulations. 

## Acknowledgment 

The guideline, authors want to thank Dr. Sonja Mathes, Dr. Jaron Zuberbier, Wiebke Filsinger and Nadine Zeyen for translation and editorial support. In addition, the authors thank Prof. Dr. Vera Mahler (Paul-Ehrlich-Institut, Langen, Germany) for helpful comments regarding the section “Allergentest solutions” as well as Dr. Helmut Sitter for his support and moderation (in line with regulations of the German Association of the Scientific Medical Societies (AWMF)). 

## Funding 

The digital conferences (Go to Training) and the AWMF moderator were financed by the DGAKI. 

## Conflict of interest 

The conflicts of interest were recorded using the AWMF portal interessenerklaerung-online.de, evaluated by the conflict of interest officer of the DGAKI (for details see the guideline report) and tabulated in accordance with the AWMF. The guideline report and conflict of interest table are available at www.awmf.org/leitlinien/detail/ll/061-009.html. 

**Figure 1 Figure1:**
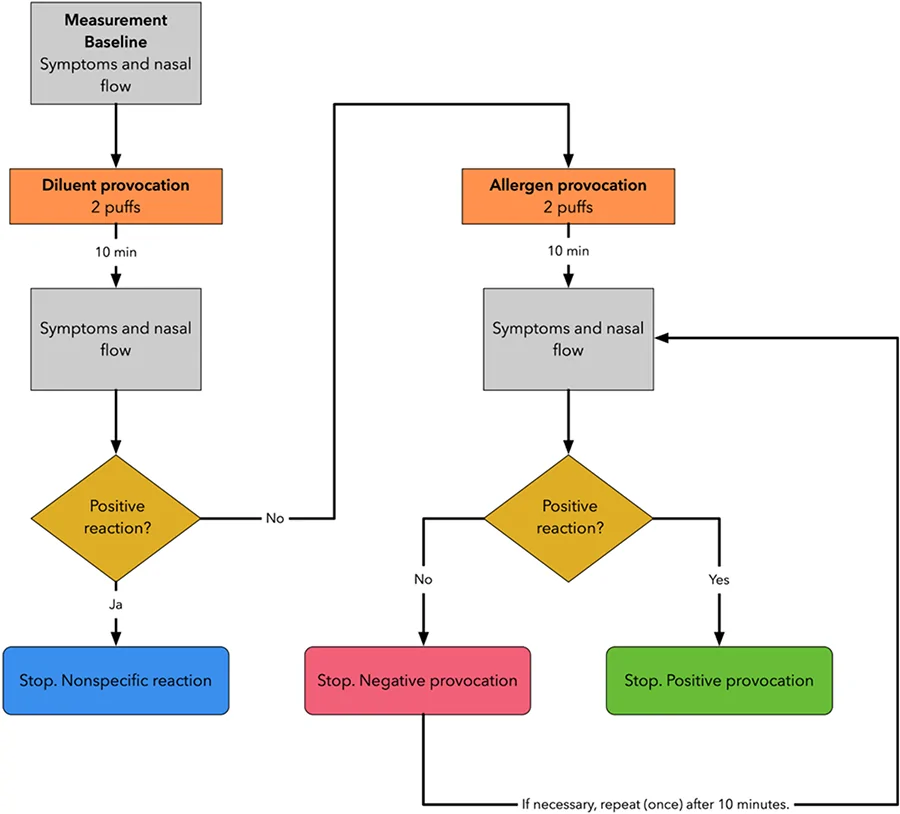
Procedure of nasal provocation test (NPT).

**Figure 2:**
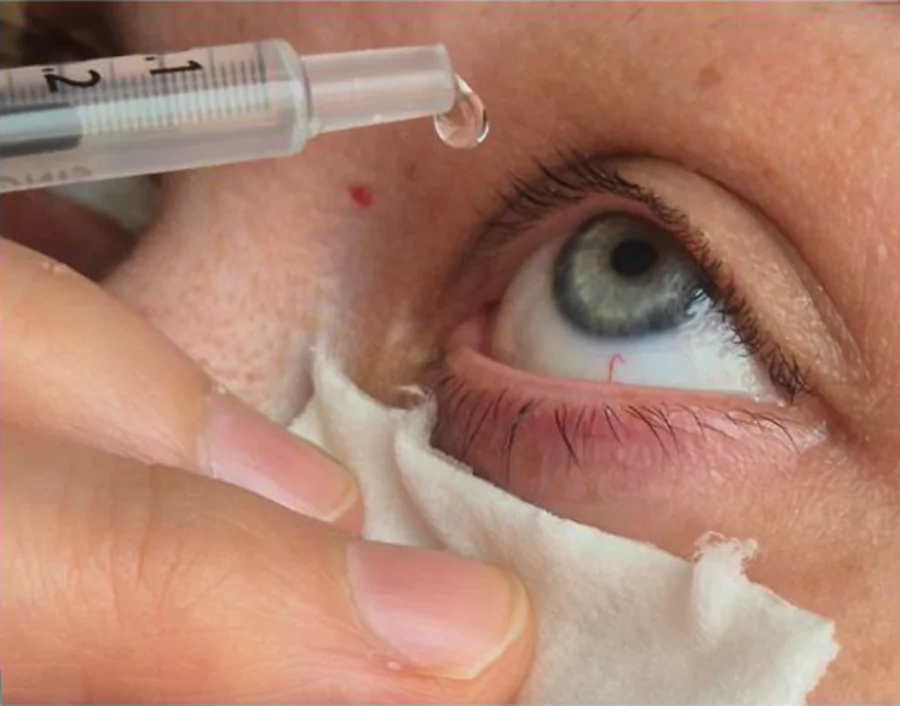
Application of the test substance to the eye (Figure Copyright M. Cuevas, Dresden).

**Table 1.** Presentation of the assessment criteria for the CPT [43]. The CPT is assessed as positive if there is a score difference of ≥ 6 between both eyes.

Itching
0 = Not itchy; 1 = Slightly itchy but not bothersome; 2 = Itchy, bothersome, and/or urge to rub eye at times; 3 = Very itchy, very bothersome, and/or urge to rub eye often.
Irritation
0 = Not irritated; 1 = Dry eye or other unusual feeling in eye, but not bothersome; 2 = Gritty or foreign object feeling in eye, bothersome, and/or urge to rub eye; 3 = Burning or painful eye, very bothersome, and/or difficulty keeping eye open, and/or urge to rub eye often.
Tearing
0 = Not watery; 1 = Watery eye, but no tears and not bothersome; 2 = Watery eye with a few tears, bothersome, and/or urge to wipe eyes at times; 3 = Watery eye with several tears, very bothersome, and/or urge to wipe eye often.
Redness
0 = No redness; 1 = Redness in conjunctiva bulbi; 2 = Redness in conjunctiva bulbi and tarsi; 3 = Redness in conjunctiva bulbi and tarsi and edema.

**Figure 3:**
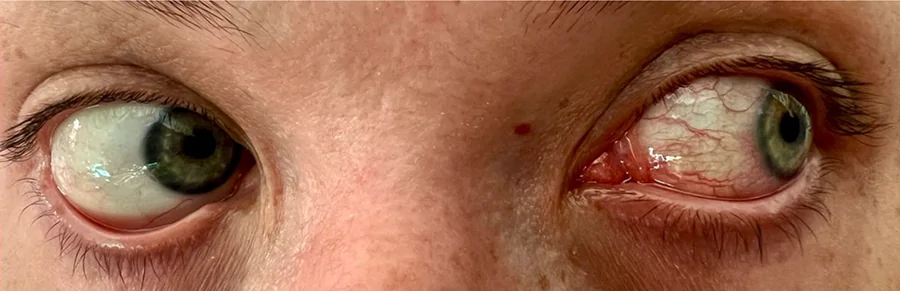
Positive reaction following application of the test substance (patient example following application of dermatophagoides pteronyssinus). Copyright M. Cuevas, Dresden.

**Figure 4 Figure4:**
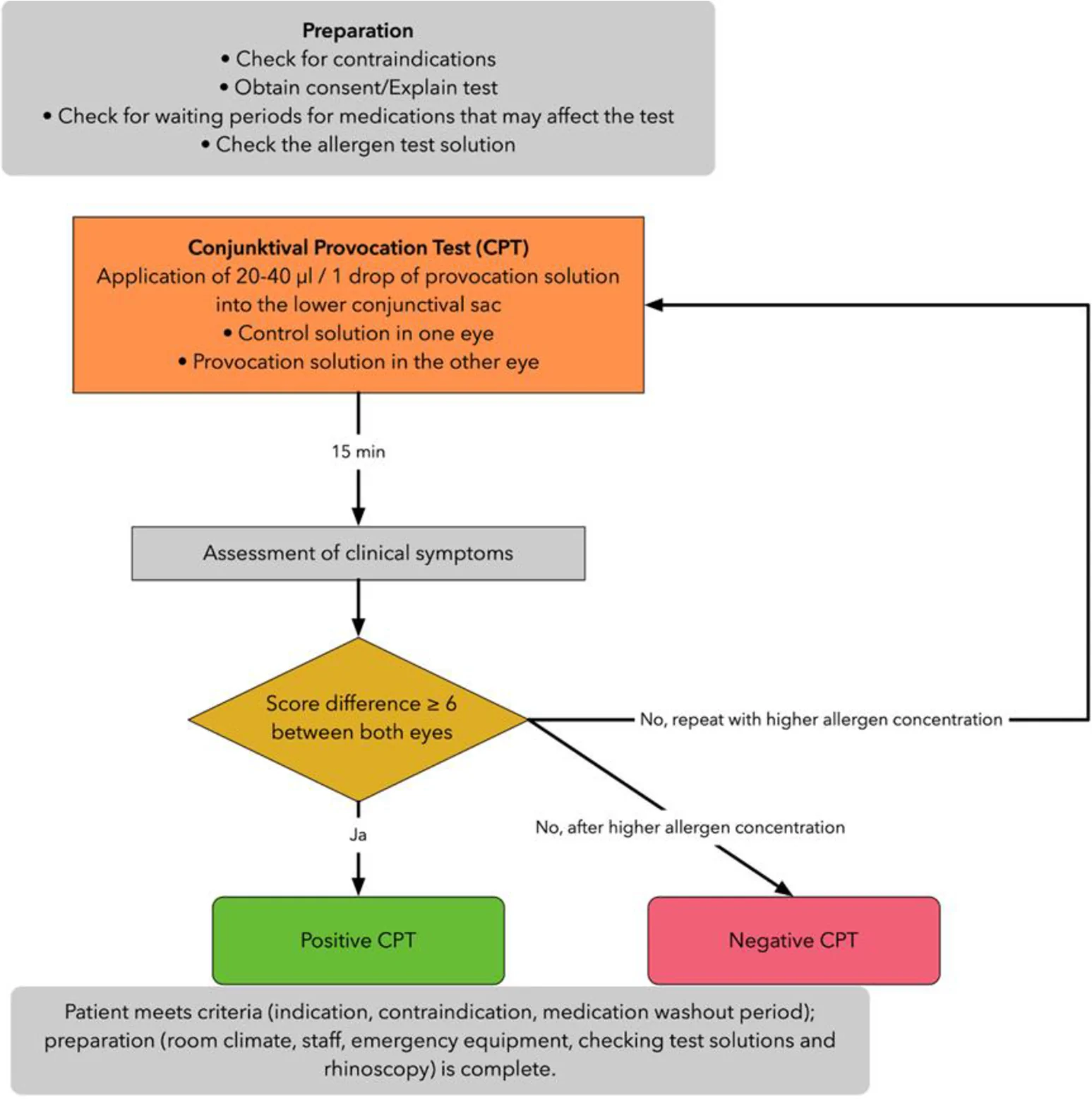
Procedure for the conjunctival provocation test (CPT).

**Table 2.** Drug withdrawal periods prior to provocation testing.

**Medication**	**Withdrawal period, oral**	**Withdrawal period, local (nasal/conjunctival)**
Mast cell stabilizers	3 d	
Corticosteroids	7 – 14 d	2 – 7 d
Antihistamines	3 – 7 d	
α-Adrenergic agents	–	1 d
Tricyclic psychotropic drugs	3 d	
NSAIDs	–	7 d
Calcineurin inhibitors (cyclosporine A, tacrolimus)	–	7 d
Montelukast	2 d	–

*Due to the limited quality of evidence regarding drug withdrawal periods, the time intervals given only represent guideline values; however, in the authors’ experience, they have proven to be effective in clinical practice [19, 37]; (d = days; NSAIDS = non-steroidal anti-inflammatory drug).

**Figure 5:**
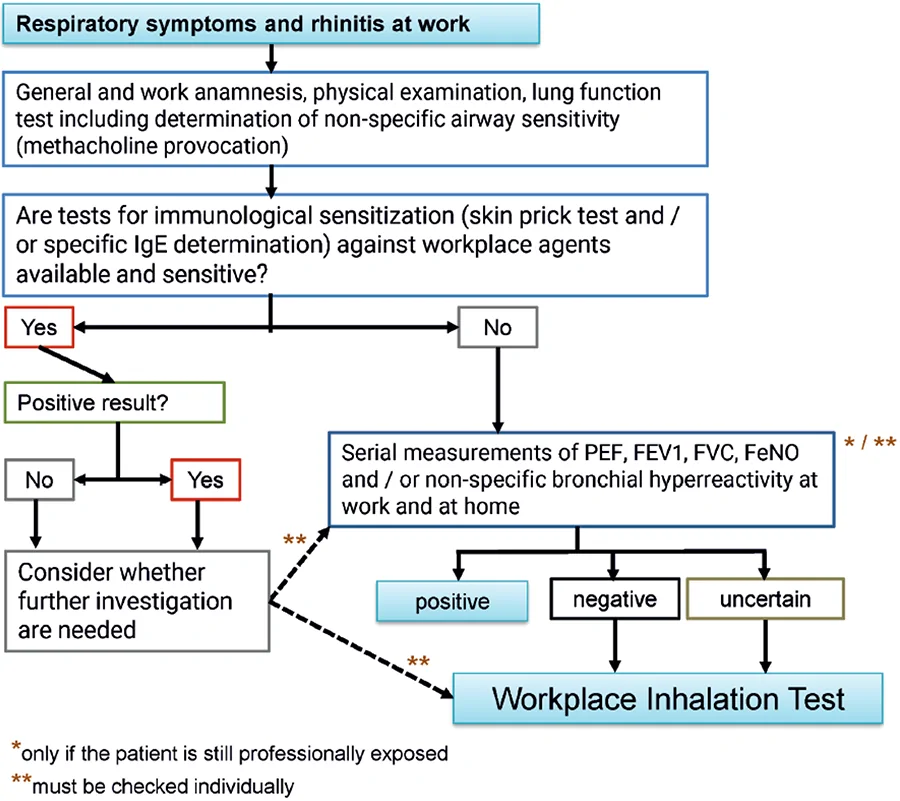
Importance of workplace exposure testing in the diagnosis of workplace-related allergic respiratory diseases [94] with kind permission of Dustri-Verlag Dr. Karl Feistle.

**Table 3.** Activities and exposures with a particular risk of developing occupational respiratory allergies.

**Substance**	**Exposure examples**
Animal and human materials	
Hair dust and dandruff from humans and animals	Hairdressing salons, agriculture, laboratories, veterinary medicine, animal farms, zoology
Bird feathers	Pet shops, poultry farms, feather processing
Mouse and rat urine	Animal care, research laboratories
Insects	Biology laboratories
House dust and storage mites	Agriculture, animal feed
Bee mites	Bee keeping
Flies, cockroaches, grasshoppers, mealworms, meal moths, rice weevils	Research laboratories, breeding operations, flour processing, feed and food industry
Chironomids	Ornamental fish feed
Bees	Beekeeping
Red spider mites	Fruit growing
Plant materials	
Flour, bran	Bakeries, pastry shops, mills
Grain dust	Agriculture, mills
Shrub and flower pollen	Nurseries
Tobacco leaves, tea	Cultivation, processing
Green coffee beans, cocoa beans, castor beans	Plantations, dock and warehouse work
Henna	Hairdressing
Molds	Waste management, agriculture, horticulture, air conditioning and humidification systems
Ficus	Horticulture, houseplants, decoration
Latex	Care and health professions, gloves
Wood dust	Carpentry, woodworking
Biological enzymes	
Amylase, xylanase, cellulase	„Flour professions“
Proteases and other enzymes	Detergent production, kitchen operations
Papain, subtilisin, pancreatin, trypsin	Laboratories, pharmaceutical companies
Pectinase	Fruit processing
Low molecular weight substances	
Isocyanates	Foam production, paints, adhesives, fillers and sealants, hardeners
Persulfates	Hairdressers, hair bleaching agents
Platinum salts	Precious metal cutting, catalyst production
Aldehydes	Disinfectants
Acid anhydrides	Plastics, synthetic resins, hardeners
Methyl methacrylates	Dentures, bone cement, artificial fingernails, adhesives
Epoxy resins	Sealing compounds, synthetic resins, paints, adhesives
Colophony	Soldering

**Figure 6:**
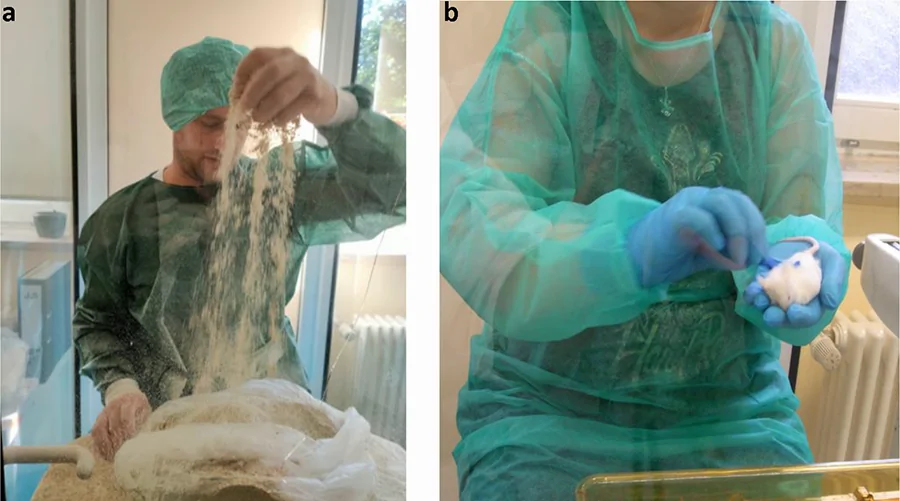
Workplace-related exposure tests with a) flour (scattering flour brought to the workplace), b) mouse epithelium (shaving a dead laboratory mouse). Copyright D. Nowak, München.

## Supplemantal meterial

Supplemental material Supplemental figure
