# Supplementary material for: S2k-Guideline – Standardized application of the nasal and conjunctival provocation test on allergic diseases of the upper airways: of the German Society for Allergology and Clinical Immunology (DGAKI), German Society of Oto-Rhino-Laryngology, Head and Neck Surgery (DGHNO-KHC), German Dermatological Society (DDG), German Society of Pediatrics and Adolescent Medicine (DGKJ), German Society of Ophthalmology (DOG), German Respiratory Society (DGP), German Society for Occupational and Environmental Medicine (DGAUM), German Society for Pediatric Allergology and Environmental Medicine (GPA), German Society for Pediatric Pulmonology (GPP), Ärzteverband Deutscher Allergologen (AeDA), and German Professional Association of Otorhinolaryngologists (BVHNO)
Source: Allergol Select. 2026 Sep 22;10:234–58. doi: 10.5414/ALX02647E (PMC13621271; doi:10.5414/ALX02647E)
Supplement: Supplementary file 1 [file allergologieselect-10-234-S01.pdf]

Documentation sheet nasal provocation test
(S2k Guideline Standardized Implementation of the Nasal and Conjunctival Provocation Test for Allergic Diseases of the Upper Respiratory Tract)

Allergen: ____________ **Patient:** ____________

Date: ____________

Examiner: ____________

BASELINE MEASUREMENT:

Symptoms (VAS scores):
Runny nose: Min ———————————————— Max
Nasal congestion: Min ———————————————— Max
Itching/sneezing: Min ———————————————— Max

Sum of VAS scores (tVAS): __________

Nasal flow: ______________________

SOLVENT APPLICATION:

Symptoms (VAS scores):
Runny nose: Min ———————————————— Max
Nasal congestion: Min ———————————————— Max
Itching/sneezing: Min ———————————————— Max

Sum of VAS scores (tVAS):____________

Nasal flow: ________________________

ALLERGEN APPLICATION:

Symptoms (VAS scores):
Runny nose: Min ———————————————— Max
Nasal congestion: Min ———————————————— Max
Itching/sneezing: Min ———————————————— Max

Sum of VAS scores (tVAS):________________

Nasal flow: ________________________

If necessary, 2nd measurement after 10 minutes without reapplying allergens
Runny nose: Min ———————————————— Max
Nasal congestion: Min ———————————————— Max
Itching/sneezing: Min ———————————————— Max

Sum of VAS scores (tVAS): _____________

Nasal flow: _________________________

RESULT:

PROCEDERE:

Limits for the evaluation of the sum of the three VAS values (tVAS):
severe symptom increase ≥120 mm, moderate symptom increase ≥95 mm;

Definition of positive nasal provocation test:
Strong reaction: drop in nasal flow > 40% OR sharp increase in symptoms tVAS ≥120 mm;
Moderate response: Drop in nasal flow ≥ 20% AND moderate increase in symptoms tVAS ≥ 95 mm;

Assessment of asthma control for adults
(National clinical Guideline for Asthma) [106]

Degrees of asthma symptom control	Well controlled	Partially controlled	Uncontrolled
Has the patient had in the last 4 weeks:<br>1. Symptoms more often than twice a week during the day<br>2. Nocturnal awakenings due to asthma.<br>3. Use of on-demand medication for symptoms more often than twice a week.<br>4. Activity restriction due to asthma	No criterion met	1 – 2 criteria met	3 – 4 criteria met

Assessment of asthma control for children and adolescents (National clinical Guideline for Asthma) [106]

Degrees of asthma symptom control	Well controlled	Partially controlled	Uncontrolled
Has the patient had in the last 4 weeks:<br>1. Symptoms during the day<br>2. Nocturnal awakenings due to asthma.<br>3. Use of on-demand medication<br>4. Activity restriction due to asthma	No criterion met	1 – 2 criteria met	3 – 4 criteria met

Lebel score [75]

Symptoms	Severity	Score
Sneezing	3 – 4<br>> 5	1<br>3
Rhinorrhea	Anterior and moderate<br>Posterior<br>Anterior and posterior<br>Highly anterior and posterior	1<br>1<br>2<br>3
Itching	Nose<br>Palate or ear	1<br>1
Nasal breathing obstruction	No nasal breathing obstruction<br>Difficult nasal breathing<br>One side of the nose is blocked<br>Both sides of the nose are blocked	0<br>1<br>2<br>3
Eye discomfort	Conjunctivitis	1

Positive rating with a score ≥ 5.

Rhinometric methods

The diagnosis of the nose includes the assessment of the respiratory performance, which is directly given by the air-volume flow. So far, 3 methods have been established internationally for the objective measurement of nasal volume flow: active anterior rhinomanometry (AAR), acoustic rhinometry (AR), and 4 phase rhinomanometry (4 PR) [107].

AAR is widely recognized as the standard method for objective assessment of the nasal airway. Other rhinologically relevant parameters can be calculated from the values of the AAR (rhinoresistometry (RRM). AR is an established method, which measures various parameters such as the minimal-cross-sectional-area (MCA) and can be seen as complementary to AAR. The 4 PR also provides supplementary information and uses an elaborate mathematical-physical analysis to calculate the entire respiratory curve and is useful as a method for scientific questions [108]. In addition, long-term rhinoflowmetry was developed for the long-term recording of nasal breathing. The measurement of peak nasal inspiratory flow (PNIF), which has been increasingly used in recent years, is a simple but also inaccurate method that measures nasal patency at maximum inspiration. In NPT, rhinomanometry is used to objectify allergen-related nasal obstruction. The sensitivity of NPT is significantly increased by rhinomanometric control compared to solely clinically controlled nasal provocation [109, 110].

1. Active anterior rhinomanometry (AAR):

Since the findings of rhinomanometry have proven to be reproducible in numerous studies [111, 112], active anterior rhinomanometry has long been considered the method of choice for quantifying nasal obstruction [113, 114] and now also has become the method of choice in the testing of aeroallergens [30, 52, 115, 116, 117].

Measurement methods:

In rhinomanometry, two quantities are recorded: the transnasal pressure difference (Δp) and the nasal volume flow ($\underline{\dot{V}}$). Pressure is defined as the force (mass × acceleration) per unit area, which is 1 Newton/m^2 = 1 Pa. In **anterior rhinomanometry,** the pressure in the choane is measured with a pressure probe in **one** nostril. This creates a closed tube system on this side, in which the pressure spreads evenly from the choane to the nostril. With the help of a pressure gauge, the difference to the external air pressure is calculated and displayed as transnasal pressure in Pa.

Breathing is done through the contralateral side, which therefore is an open tube system. Accordingly, the pressure from the choane to the entrance of the nose drops to the ambient pressure (set as 0). On this side, the volume flow rate is measured in cm^3/s (mL/s). To do this, the patient must breathe in and out normally through a breathing mask with their mouth closed. The breathing air flows through a pinhole aperture (or a butcher's nozzle) into the outer space. Here, the pressure is measured in front of and behind the pinhole and the volume flow is determined via the calculated pressure drop according to the formula $\Delta p = \frac{\Delta V^2}{2A^2}$ (A = area of the pinhole).

Curves:

The presentation of the measured values for volume flow and pressure difference was specified in Standardization Guidelines 1984 [118] and are considered an international standard. They are recorded in an XY system as follows: Inspiration right (1st quadrant: right side of the nose, 2nd quadrant: left side of the nose), expiration left (3rd quadrant: left side of the nose, 4th quadrant: right side of the nose) [113]. At small volume flows, the flow is laminar, so that the transnasal pressure is proportional to the volume flow. Δp = R * V (R = flow resistance). Normally, this laminar area is less than 20 cm3/s. Otherwise turbulent flow occurs, meaning that the transnasal pressure is approximately proportional to the square of the volume flow: Δp ~ R * V2. Therefore, the nasal airway resistance is not constant, but changes constantly during breathing. In order to be able to illustrate these complicated conditions clearly and to make a comparison between different curves, the V_{150} parameter is described. V_{150} specifies the volume flow in cm^3/s at a transnasal pressure of 150 pascals (Pa) [119].

Normal values:

The average nasal volume flow of the total nose at a transnasal pressure difference of 150 Pa (V_{150}) is 670 + 160 cm^3/s, after deswelling of the nasal mucosa with alpha-adrenergic substances the difference is 810 + 170 cm^3/s. With restrictions, a table for assessing the nasal volume flow can be derived from the standard values of the volume flow of both sides of the nose [120]: <u>V_{150} (cm^3/s)</u>

0 - 200: insufficient

200 - 400: severe obstruction

400 - 500: mild obstruction

500 – 1.000: normal breathing

Nasal cycle:

Knowledge of the nasal cycle is important for the assessment. The cycle duration is variable, in general it is between 2 - 6 hours depending on the individual. The cause seems to be an alternation between sympathetic and parasympathetic stimulation – controlled by the hypothalamus. While the mucous membrane of one side of the nose swells, the contralateral side of the nose swells up. If both sides swell up, airflow will be reduced. The air flow of the nose remains fairly constant during this change [114]. This is called the classic nasal cycle, which is present in about 80% of the population. The side with a high air flow rate is in the working phase: the air is heated and moistened to prepare it for entry into the lungs [121]. The swollen side is in the resting phase, the mucous membrane absorbs moisture and establishes heat again.

Other authors distinguish between four additional nasal cycles [122]:

- The inconcert type: Rise and fall of the air flow in both sides of the nose equally.
- The one-sided type: swelling and decongestion only on one side of the nose, the opposite side does not change.
- The non-cycle type: Both sides of the nose do not change their swelling state.
- The mixed type: The different types of nasal cycle occur distributed during the day and night.

Kern [123] and Mlynski [124] already recognized the complexity of cyclic nasal mucosal swelling. It was only with the help of long-term rhinometry that the five different nasal cycle types could be identified [125].

Errors:

Typical errors occurring in rhinomanometry can be identified with the following: a leaking breathing mask, a leaking pressure probe and/or mouth breathing during the measurement. If the used nose adapters are too large, the mutual nostril will be deformed. This results in false low values. If there is a nasal septum perforation, there are no usable results [126].

2. Rhinoresistometry (RRM):

Mlynski developed rhinoresistometry (RRM) in 1993 [127, 128], whereby rhinologically relevant parameters are calculated from the values of the AAR, allowing the examination procedure and the measurement to be completely in line with the AAR. Based on the RRM, the flow resistance of the nose, the turbulence behavior, the hydraulic diameter, and the coefficient of friction (configuration of the inner nose) can be displayed. This makes it

possible to identify causative pathologies for nasal obstruction, such as constriction, nasal alar collapse, or pathological turbulences.

3. Acoustic rhinometry (AR):

Acoustic rhinometry is based on the physical principle that the impedance of a tube for a sound pressure wave (over 250 Hz) depends on local changes in the geometry [129].
By analyzing the amplitude of the sound waves of structures in the nasal cavity, an estimate of the intranasal geometry can therefore be made with software as a diagram of the cross-sectional area against the distance from the nostril. In this diagram, constrictions are shown as depressions and extensions as peaks [130, 131].
The minimum cross-sectional area of the nose, the cross-sectional areas at certain distances, and the volumes of certain parts of the nasal cavity can be calculated.
A sound pulse of 150-10,000 Hz is directed into the main nasal cavity via a nasal applicator [132, 133].
After reflection on the endonasal structures, the sound is detected by a microphone. Multiple readings are taken from each side of the nose and averaged.
Acoustic rhinometry is fast, reproducible, non-invasive, requires minimal patient cooperation, and can therefore be used even in young children [134, 135, 136].
It provides two-dimensional information, does not require transnasal airflow, and indicates the presence of nasal obstruction as well as locating the site of the obstruction.
Acoustic rhinometry was used for allergen provocations [77, 137, 138, 139, 140], histamine, bradykinin and methacholine provocation tests [141, 142, 143], provocation tests with cold, dry air [144], but also for ASA provocation tests [145, 146] and nasal provocation testing for other forms of non-allergic and occupational rhinitis [74, 147, 148, 149].

4. 4-phase rhinometry (4PR):

4-phase rhinomanometry, like classical rhinomanometry, measures nasal breathing as inspiration and expiration. However, inspiration and expiration are both divided into an accelerating and a decelerating part - a total of 4 phases.
By computerized measurement of these phases, a vertex resistance (VR) can be calculated over the entire inspiration and expiration, which also includes the total volume of nasal breathing. In addition, effective resistance (reef) can be added as a summation of quick measurements for inspiration and expiration as an additional parameter [150].

VR and Reff partially describe the turbulent and laminar flow in the same breath and show the hysteresis of the rhinomanometric curve in the ascending and descending graph of the coordinate system. The loops during inspiration and expiration (hysteresis) are caused by the nasal anatomy, the elasticity of the nasal wall, and the Bernoulli effect of the nasal valve. This results in typical curves for the corresponding clinical symptoms [151]. In contrast to classic rhinomanometry, 4PR uses VR and Reff to measure the entire hysteresis curve in the ascending and descending graphs, rather than just the average flow value at 150 pascals.

The relationship between pressure and flow of nasal breathing is not described by a point in the coordinate system at 150 pascals, but by areas below the respiratory curve. VR and Reff correlate well with each other and show only minor differences. However, it could be shown that the logarithm of VR and Reff is similar to a normal distribution and that the obstruction can therefore be more easily demonstrated. It could also be demonstrated that the subjectively perceived restriction of nasal breathing on a logarithmized visual analogue scale (VAS) correlated better with the logarithm of VR (LVR) and Reff (LER) in test subjects. LER showed the highest correlation between resistance and subjective assessment by the patients [151].

The information about the flow at a given pressure provides only limited significance for the critical analysis of nasal breathing, especially when it comes to scientific questions. The 4-phase rhinomanometry is more difficult to assess due to the many parameters and the complex mathematical background but is useful for scientific questions due to its complexity.

5. Long-term rhinoflowmetry (LRM):

The methods mentioned (AAR, RRM, and AR) only determine the state of nasal breathing at the time of the examination. A recording of the nasal cycle and disturbances of the nasal flow in everyday life and at night is therefore not given. Long-term rhinoflowmetry (LRM) was developed for the long-term recording of nasal breathing. The group led by G. Mlynski published their findings regarding LRM for the first time as a new method for functional nasal diagnostics [152]. It consists of a small portable device and a nasal cannula. The course of the nasal volume flow as well as the heart and respiratory rate and the nasal minute volume are recorded separately.

Now it was possible to continuously measure the volume flow of the nose over 24 hours and more, even during physical activity and sleep.

This made it possible to better grasp the complexity of the nasal cycle, in particular to work out the 5 different types of the nasal cycles. Since physical activity, body position, sleep as well as nasal allergies change the nasal cycle, this method allows clinicians to draw conclusions about the drug influence on the nasal mucosa over time.
However, the number of examinations using LRM is still low [153, 154, 155].

6. Peak nasal inspiratory flow (PNIF):

Measuring peak nasal inspiratory flow (PNIF) is a simple and less complex way to measure nasal flow at maximum inspiration [30]. PNIF measurements are taken bilaterally.
A conventional inspirational flowmeter is used for this purpose. The patient inhales 3 times to the maximum. Only the highest value of these three measurements in liters/minute is used for the evaluation. Fuller et al. [156] compared these results with the NOSE scale (Nasal obstruction symptom score evaluation) [157, 158]. After functional septorhinoplasty, the NOSE scale values decreased statistically significantly postoperatively ($p< 0.001$). The measured PNIF values increased in line with the increased air volume flow rate achieved by the operation.
The volume changes measured pre- and postoperatively were significant ($p< =0.001$). However, when comparing the NOSE scales and the PNIF results, this resulted in a weak correlation ($r=-0.26$; $p=0.02$). This would be necessary to establish PNIF as an objective and accurate method for measuring the air volume flow of the nose.
A certain inaccuracy can probably be explained by the measurement method as such. At maximum inspiration, the nostrils stand inwards into the clear width of the nose and throttle the air supply. In the case of soft cartilage of the nostrils, partial or complete nasal alar collapse on one or both sides is also possible, which massively restricts breathing in the area of the nasal valve. With maximum exhalation through the nose, the nostrils cannot collapse.
This is adopted by the FEV1 nose method, which was published earlier on for the intranasal provocation test [159]. This method is also strongly dependent on the expiratory performance of the patient, just as the PNIF method is dependent on the inspiratory performance.
Of course, the strength of the inspirational performance cannot always be the same. This explains the inaccuracy of the PNIF method. For the extent of nasal obstruction, it is therefore more suitable from an informative point of view.

Due to its simple technical implementation, PNIF measurement has found international acceptance as a method of outcome measurement of nasal allergen provocation [30, 52, 160, 161, 162].

Conclusion

Both AAR and the PNIF are applied as standard procedures for assessing the outcome of the NPT in Germany. In individual cases, other rhinometric methods may also be used.

Recommendation

One of the two methods mentioned above, AAR or PNIF, should be used for the assessment of nasal flow in the NPT (consensus: agreement of 92%).
